# Advances in Diversity, Evolutionary Dynamics and Biotechnological Potential of Restriction-Modification Systems

**DOI:** 10.3390/microorganisms13051126

**Published:** 2025-05-14

**Authors:** Chen Chen, Yue Zhang, Hao Wu, Jianjun Qiao, Qinggele Caiyin

**Affiliations:** 1 School of Chemical Engineering and Technology, Tianjin University, Tianjin 300072, China; cc_2019@tju.edu.cn (C.C.); 16602659785@163.com (Y.Z.); jianjunq@tju.edu.cn (J.Q.); 2 State Key Laboratory of Synthetic Biology, Tianjin University, Tianjin 300072, China; 3 Zhejiang Institute of Tianjin University (Shaoxing), Shaoxing 312300, China; dream72wh@tju.edu.cn

**Keywords:** restriction–modification systems, microbial immunity, genomic stability, biotechnology, evolutionary dynamics, microbial genetics

## Abstract

Restriction–modification systems (RMS) are ubiquitous in prokaryotes and serve as primitive immune-like mechanisms that safeguard microbial genomes against foreign genetic elements. Beyond their well-known role in sequence-specific defense, RMS also contribute significantly to genomic stability, drive evolutionary processes, and mitigate the deleterious effects of mutations. This review provides a comprehensive synthesis of current insights into RMS, emphasizing their structural and functional diversity, ecological and evolutionary roles, and expanding applications in biotechnology. By integrating recent advances with an analysis of persisting challenges, we highlight the critical contributions of RMS to both fundamental microbiology and practical applications in biomedicine and industrial biotechnology. Furthermore, we discuss emerging research directions in RMS, particularly in light of novel technologies and the increasing importance of microbial genetics in addressing global health and environmental issues.

## 1. Introduction

Restriction–modification systems (RMS) are fundamental adaptive mechanisms in microbial genetics, providing crucial defense against exogenous genetic threats and playing a pivotal role in microbial genomic evolution. These systems, which encompass both restriction enzymes and modification enzymes, have been instrumental in shaping our understanding of microbial genomic integrity and have had profound implications for both evolutionary biology and molecular biotechnology. The discovery of restriction enzymes by Arber, Smith, and Wilcox in the early 1960s and 1970s marked a transformative moment in molecular genetics, offering the first reliable tools for manipulating DNA [1,2]. This breakthrough also unveiled an intricate array of microbial protective strategies against phages and plasmids, significantly advancing our knowledge of microbial defense systems and their roles in genomic stability [3].

The interplay between restriction enzymes, which recognize and cleave specific DNA sequences, and modification enzymes, which methylate host DNA to protect it from cleavage, exemplifies a sophisticated biological mechanism that distinguishes between self and non-self [4,5]. This system, which serves as an advanced immune-like defense and regulatory mechanism, has evolved over millennia, reflecting a highly refined biological process that underpins microbial survival and adaptation [6]. Recent advancements in next-generation sequencing technologies have illuminated the vast diversity of RMS across microbial genomes, revealing not only their pivotal role in microbial defense but also their broader contribution to microbial adaptability [7]. Such discoveries highlight the untapped potential of RMS for a range of biotechnological applications, from genetic engineering to antimicrobial development [8]. The significance of RMS extends far beyond basic molecular biology. Restriction enzymes, as essential tools in gene cloning, DNA mapping, and genomic editing, have been at the heart of revolutionary advances in molecular genetics [9]. Their precision and versatility have enabled the development of advanced genetic manipulation techniques, including CRISPR-based technologies, which share roots in microbial adaptive immune-like defense systems similar to RMS [10]. Furthermore, understanding RMS is crucial for addressing challenges in antimicrobial resistance, as these systems play vital roles in horizontal gene transfer and microbial competition, mechanisms that are key to the spread of resistance genes in pathogenic bacteria [11,12,13,14].

The ecological and evolutionary roles of RMS further enhance their relevance, providing critical insights into microbial community dynamics, pathogen-host interactions, and the mechanisms by which microbes resist phages [15]. These dynamics are of particular importance in biotechnology, environmental biology, and healthcare, where microbial interactions influence diverse processes, from fermentation to infectious disease transmission [16]. The impact of RMS on microbial ecology is also evident in their influence on the evolutionary strategies of prokaryotic communities. Studies on pathogens such as *Staphylococcus aureus*, *Bacillus anthracis*, and *Campylobacter coli* have shed light on the ways in which RMS affect pathogenicity, virulence, and host immune evasion [17]. Mechanisms such as phase variation and molecular mimicry, mediated by RMS, contribute to microbial diversity and provide a selective advantage in a variety of ecological contexts.

The role of RMS in microbial defense represents an ongoing “arms race” between bacteriophages and bacterial hosts [18]. While originally identified for their ability to cleave invading DNA in a sequence-specific manner, RMS have recently been implicated in a wider array of biological processes, including microbial evolution, epigenetics, and gene expression regulation [19]. Quantitative models of RMS have revealed the regulatory complexities of these systems, elucidating how they balance the dual imperatives of defending against invasive genetic elements while avoiding self-damage [20]. Furthermore, the pervasive presence of diverse antiphage defense systems across the microbial pangenome underscores the central role of RMS in microbial survival and evolution [21,22].

**Figure 1:**
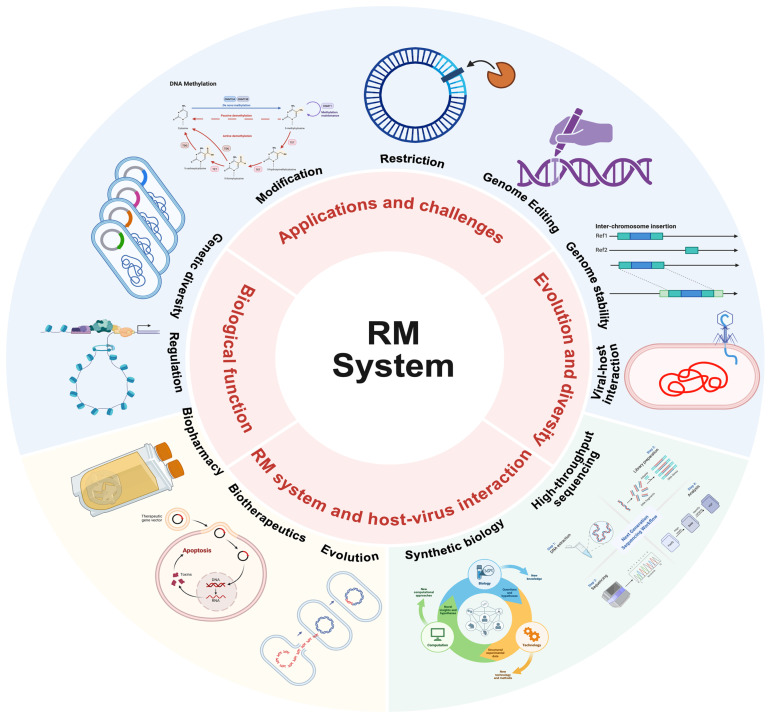
Roles of RM systems in various fields.

This review addresses not only the functional diversity and biotechnological applications of RMS but also comprehensively discusses structural insights gained from recent crystallographic and cryo-electron microscopy studies, and elucidates the evolutionary mechanisms underlying the remarkable diversification of RMS enzyme families across prokaryotes, which aims to explore the multifaceted roles of RMS in microbial biology, biotechnology, and medicine. It will highlight recent advancements in our understanding of RMS, address current challenges in the field, and offer insights into their biochemical foundations, as well as their profound impacts on microbial ecology and evolution (Figure 1). By synthesizing current knowledge, this review will provide a comprehensive overview of RMS, preparing the ground for more detailed investigations of these adaptable molecular mechanisms.

## 2. Classification and Diversity of RMS

The intricate operations of RMS in microorganisms epitomize a sophistical mechanism of genomic defense and regulation, pivotal for microbial survival and evolutionary dynamics. These systems comprise two core components: restriction enzymes (REases) and modification enzymes (MTases), each playing a distinct yet complementary role in DNA recognition and processing (Table 1).

### 2.1. Structural Characteristics of RMS

#### 2.1.1. Restriction Enzymes

**Figure 2:**
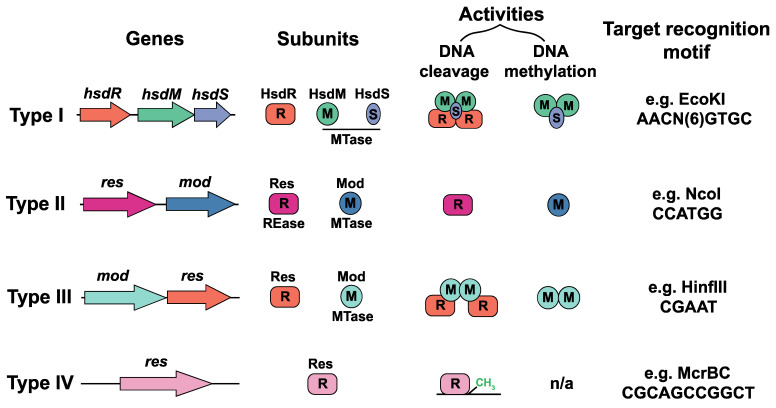
Schematic representation of the classification and mechanisms of RM systems. This diagram illustrates the four major types of RM systems (Types I–IV) and highlights key mechanistic features. Type I systems: Multi-subunit complexes (HsdR, HsdM, HsdS) that require ATP and SAM, with distant cleavage from recognition sites. Type II systems: Single-subunit enzymes that cleave at or adjacent to the recognition sequence without ATP requirement. Type III systems: Heterotetrameric complexes (Res and Mod) that need ATP for activation and cleave DNA ~25 bp away from the recognition site. Type IV systems: Enzymes such as McrBC that target methylated DNA using GTP as a cofactor. n/a, none.

REases are a diverse group of enzymes that safeguard bacterial cells from foreign DNA infiltration, such as that from bacteriophages or plasmids. These enzymes act by scanning the DNA for specific sequences, known as recognition sites, and cleaving the DNA at or near these sites. Restriction enzymes are categorized into four major types—Type I, II, III, and IV—based on their structure, recognition sequence specificity, and cleavage position relative to the recognition site (Figure 2) [27].

Type I restriction enzymes are multifunctional complexes that catalyze both DNA restriction and modification. Unlike Type II enzymes that cleave at defined sites near the recognition sequence, Type I enzymes recognize specific DNA sequences but cleave at distant sites, sometimes thousands of base pairs away. A typical Type I system comprises three subunits: HsdR (restriction), HsdM (modification), and HsdS (specificity) [28]. Their activity requires ATP and S-adenosyl-L-methionine (SAM) for DNA modification and cleavage. Upon target recognition, the enzyme undergoes ATP-driven conformational changes and translocates along the DNA, eventually cleaving both strands at a remote, random location. Although their unpredictable cleavage patterns pose challenges for conventional molecular cloning [29]. Type I enzymes have been exploited in applications where such randomness is advantageous. For instance, in genome editing and molecular evolution, they have been used to induce targeted mutations or generate gene libraries by introducing variability. Their capacity to interact with large DNA regions also makes them valuable for genome mapping and microbial strain typing, where complex DNA interaction patterns yield insights into genetic markers and diversity [23,30]. Despite limitations compared to more predictable Type II enzymes, Type I systems have found niche applications in bacterial typing and gene regulation studies. Ongoing research and optimization of these enzymes may further expand their utility in high-throughput screening and genetic analysis [9,29,31,32].

Type II restriction enzymes are indispensable tools in molecular biology, prized for their ability to cleave DNA at precise, short (4–8 bp) palindromic sequences. They cleave at fixed positions either within or adjacent to their recognition sites, producing either sticky ends—which facilitate efficient ligation in recombinant DNA technologies—or blunt ends. To date, over 3000 distinct Type II enzymes have been identified, with more than 600 commercially available from companies such as New England Biolabs (Ipswich, MA, U.S.) and Thermo Fisher (Waltham, MA, U.S.), underscoring their central role in molecular cloning, genome editing, and synthetic biology [33,34]. A key advantage of Type II enzymes is their operational simplicity. In contrast to Type I enzymes, which require ATP and multiple subunits, Type II enzymes function as monomeric proteins without the need for ATP, resulting in easier handling and higher efficiency in laboratory protocols. Iconic examples include EcoRI, which recognizes 5′-GAATTC-3′ and produces a 5′ overhang, and SmaI, which cleaves 5′-CCCGGG-3′ to yield blunt ends. Other widely used enzymes, such as BamHI (recognizing 5′-GGATCC-3′) and HindIII (recognizing 5′-AAGCTT-3′), are integral to the construction of expression vectors and the preparation of DNA libraries. Additionally, enzymes like NotI and XhoI are favored for specialized applications, such as high-copy vector cloning and high-fidelity restriction digests [35].

Type III enzymes recognize specific sequences but cleave DNA at a short distance away from their recognition sites and require ATP. Type III restriction enzymes, less commonly used in laboratory settings, are composed of two subunits: Res (restriction) and Mod (modification), which function as part of a complex. These enzymes recognize specific DNA sequences but cleave the DNA approximately 25–27 bp away from the recognition site. Type III enzymes require ATP for cleavage but, unlike Type I enzymes, do not use it for DNA translocation. Instead, ATP is involved in the activation of the cleavage mechanism. The cleavage occurs only when two enzyme complexes bind in a head-to-head orientation on opposite strands of the DNA, ensuring that cleavage happens at a precise distance from the recognition site [36].

Type IV enzymes target modified DNA, such as methylated or hydroxymethylated DNA, and are less common in RMS research, which are modifications often found on phage DNA as evasion strategies against host restriction systems [24]. These enzymes recognize specific modifications rather than specific sequences and cleave modified DNA to protect the bacterial cell from foreign genetic elements. Type IV enzymes are crucial for bacterial defense against phages that have evolved to mimic host methylation patterns to evade Type II restriction systems. An example of a Type IV enzyme is McrBC, which requires GTP for cleavage and targets DNA containing methylcytosine [37].

#### 2.1.2. Modification Enzymes

**Figure 3:**
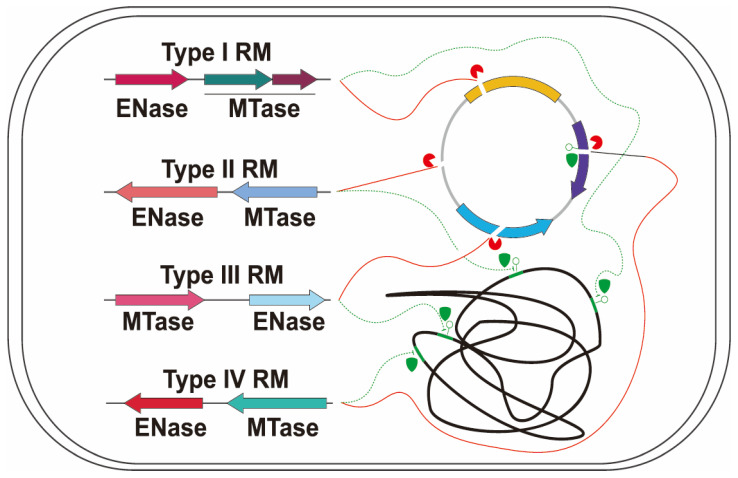
A schematic representation showing functions of RMS as immune-like defense systems in protection of microbial genomes. Restriction–modification systems (RMS) detect the methylation patterns of invading foreign DNA, such as artificial plasmids. Methylated sequences are identified as self, whereas unmethylated recognition sequences on the incoming DNA are considered non-self and are cleaved by REases. The methylation status of genomic recognition sites is preserved by the cognate MTases of RMS.

MTases play a critical role in the protection of the host genome from the action of its own restriction enzymes (Figure 3). These enzymes function by catalyzing the transfer of a methyl group from S-adenosylmethionine (SAM) to specific nucleotides—typically adenine or cytosine residues—within the host DNA. This methylation occurs at specific recognition sequences that are also targeted by the associated restriction enzymes, preventing these enzymes from recognizing and cleaving the host DNA. By modifying these key nucleotide residues, MTases effectively create a protective barrier, ensuring that the host genome remains intact while allowing restriction enzymes to target and degrade foreign DNA that lacks the protective methylation marks. The methylation pattern established by MTases serves as a molecular “self” signature, distinguishing the host’s DNA from non-methylated, foreign DNA [38]. This self/non-self discrimination is essential for the proper functioning of the RMS, which serve as an adaptive immune-like defense mechanism against the intrusion of exogenous genetic elements, such as bacteriophages and plasmids (Figure 2). In this way, MTases help to maintain genomic integrity by safeguarding the host from its own restriction enzymes while enabling the immune-like defense against foreign invaders. The enzymatic activity of MTases is highly specific to the sequence context in which the methylation occurs. In many bacteria, MTases are associated with their corresponding restriction enzymes, forming a paired system. The restriction enzyme recognizes and cleaves DNA at specific sites, while the MTase modifies the host’s DNA at these same sites to prevent cleavage [28]. This coordinated action between the restriction enzyme and MTase constitutes a highly effective defense mechanism, which has been finely tuned through evolution to ensure that microbial genomes are protected while still capable of responding to foreign genetic threats.

In addition to their protective function, MTases have significant implications for the regulation of gene expression and epigenetic modifications. By methylating specific sites within promoter regions or other regulatory sequences, MTases can influence the transcriptional activity of genes, adding another layer of complexity to microbial gene regulation. Recent studies have also revealed that DNA methylation can be dynamic, with modifications being reversible under certain conditions, suggesting that MTases may play a role in microbial adaptation to environmental stressors or changes in the host genome [39]. Furthermore, MTases are involved in a variety of other biological processes, including genome stability, DNA repair, and the regulation of mobile genetic elements. The presence of MTases can influence the ability of mobile genetic elements, such as plasmids or transposons, to insert into or recombine with the host genome, thereby contributing to the overall genetic diversity of microbial populations [40]. In this context, MTases not only serve as defenders of the genome but also play an active role in shaping microbial evolution. The diversity of MTases across different microbial species is vast, with variations in both their sequence specificity and their mode of action. Some MTases exhibit broad specificity, modifying a wide range of sequences, while others are highly selective, targeting very specific DNA motifs [41,42,43]. This diversity has profound implications for the biotechnological use of MTases in applications such as gene editing, where the precise control of DNA modifications is essential. For instance, engineered MTases can be used to introduce specific methylation marks at predetermined sites within a genome, offering a powerful tool for epigenetic manipulation and genetic modification [44].

### 2.2. Diversity of RMS

The RMS represent a crucial evolutionary adaptation in microorganisms, enabling them to combat the influx of foreign genetic elements such as bacteriophages and plasmids. The astonishing diversity of these systems across different microbial taxa underscores their important role in microbial ecology, evolution, and genetic innovation. Present across a wide array of microbial taxa, RMS are not a monolith but a mosaic of diverse, intricate systems that reflect the evolutionary history, ecological pressures, and genetic dynamism of their microbial hosts.

#### 2.2.1. Structural Diversity of REases

REases exhibit remarkable structural diversity, reflecting their evolutionary adaptation to varied functional demands. Type II restriction enzymes, which are most thoroughly studied and widely used, typically adopt homodimeric structures that recognize palindromic DNA sequences. Crystal structures of well-known Type II enzymes such as EcoRI, BamHI, and HindIII have revealed a common architecture characterized by catalytic sites involving divalent metal ions (commonly Mg^2+^), essential for phosphodiester bond cleavage [45]. These structures typically include two separate monomers interacting symmetrically with the DNA substrate, precisely positioning catalytic residues for cleavage.

In contrast, Type I restriction–modification enzymes represent large multi-subunit complexes (comprising HsdR, HsdM, and HsdS subunits), whose DNA cleavage mechanism is ATP-dependent and structurally more complex. Cryo-electron microscopy (cryo-EM) and crystallographic studies of Type I enzymes have revealed dynamic conformational changes triggered upon DNA binding, accompanied by translocation along the DNA helix, ultimately cleaving DNA at distant sites from the recognition sequences [28]. Type III restriction enzymes, structurally characterized by Res and Mod subunits, also exhibit intricate interactions, forming asymmetrical heterotetramers. These complexes require ATP to mediate cleavage at defined distances from their recognition sites. Structural studies indicate ATP hydrolysis triggers a conformational transition critical for DNA cleavage, highlighting the sophisticated coupling between nucleotide hydrolysis and restriction activity [36]. Type IV enzymes, targeting methylated DNA, display structural adaptations for recognizing DNA modifications rather than specific nucleotide sequences. For instance, the Type IV enzyme McrBC functions as a complex of two distinct proteins (McrB and McrC) and employs GTP hydrolysis for coordinated DNA cleavage [25]. Crystal structures of McrBC complexes have illuminated how protein-DNA interaction and GTPase activity integrate to discriminate methylated from unmethylated DNA. This structural diversity not only underpins the functional versatility of RMS but also provides a robust foundation for their extensive biotechnological exploitation, from molecular cloning to epigenetic studies and beyond.

#### 2.2.2. Comparative Genomics and the Landscape of RMS Diversity

Comparative genomic analyses have uncovered striking differences in RMS architecture among diverse bacterial species. For instance, a study comparing the genomes of Escherichia coli strains from different ecological niches revealed that pathogenic strains often harbor additional RMS loci, which may serve to restrict horizontal gene transfer of virulence factors [46]. Another study in Streptococcus species demonstrated that RMS loci are hotspots for genomic rearrangements, suggesting an adaptive mechanism to rapidly respond to phage predation in competitive environments [47]. These examples, together with analyses of microbial communities in extreme environments such as high-saline or high-temperature habitats, provide concrete evidence of the ecological significance and evolutionary pressures shaping RMS diversity. The diversity of RMS is characterized by a wide array of sequence specificities, functional mechanisms, and regulatory frameworks, all of which contribute to their essential role in microbial defense against foreign DNA, including bacteriophages, plasmids, and other mobile genetic elements.

One particularly striking finding from comparative genomic analyses is the identification of RMS loci as hotspots for genomic rearrangements. The identification of RMS loci as hotspots for genomic rearrangements is significant because these rearrangements underpin rapid evolutionary adaptation and genetic diversification. RMS loci frequently harbor repetitive elements, transposons, and insertion sequences, facilitating recombination events. Such rearrangements contribute not only to the rapid evolution of the RMS itself but also significantly impact surrounding genomic regions. In pathogenic bacteria such as *Neisseria meningitidis* and *Helicobacter pylori*, these rearrangements drive antigenic variation, aiding in immune evasion and enhancing virulence [48,49]. Furthermore, genomic plasticity at RMS loci influences microbial adaptability, allowing bacterial populations to respond swiftly to environmental challenges or competitive pressures. Thus, the evolutionary significance of RMS-driven genomic rearrangements extends beyond mere system diversification, profoundly affecting bacterial genome structure, ecological fitness, and evolutionary trajectories. These rearrangements, which include gene duplications, (HGT, and recombination events, underscore the importance of RMS in microbial genomic conflict and adaptation [50]. The highly variable nature of these loci suggests that RMS are not static, but instead, evolve rapidly in response to selective pressures such as phage predation, HGT, and ecological competition [51]. These findings illustrate how rapid genetic diversification at RMS loci in bacterial species such as *E. coli* and *S. pneumoniae* occurs in direct response to selective pressures exerted by evolving bacteriophage populations. Experimental evolution studies have documented how bacteria and phages continually acquire mutations, modifying RMS specificity and phage genomes, respectively, demonstrating a continuous reciprocal adaptation typical of evolutionary arms races [15,52]. The adaptation of RMS to environmental pressures involves several key mechanisms. Gene duplication and divergence are primary drivers of RMS expansion, allowing microorganisms to enhance their repertoire of restriction and modification enzymes. This process enables the evolution of new or more specific enzymes to target a broader range of foreign genetic material. HGT plays an equally significant role by facilitating the acquisition of RMS loci from other microorganisms. This process allows for the rapid incorporation of novel restriction and modification systems that may offer better protection against new threats, such as emerging phage strains or new plasmid-born genes. Additionally, recombination events within and between RMS loci enable microbial populations to rapidly adapt, creating new specificity determinants and regulatory mechanisms that better suit the selective pressures of their environments. Furthermore, the evolution of RMS is not limited to their genetic components. Regulatory adaptation also contributes to the expansion of RMS diversity. As microbial genomes encounter shifting environmental conditions, such as fluctuating phage populations or changing availability of genetic material through HGT, the regulation of RMS expression is often modified to enhance protection while minimizing energy costs. This dynamic regulation of RMS ensures that they are expressed when necessary but remain dormant when the threat of foreign DNA is minimal. These adaptive regulatory strategies include the modulation of transcriptional activators and repressors, which adjust the expression of RMS loci in response to environmental signals like the presence of phage or other mobile genetic elements.

A key insight from these comparative genomic studies is the correlation between the complexity of RMS and the ecological niche or lifestyle of the organism. For example, microorganisms that experience high levels of genetic exchange, such as soil-dwelling bacteria or members of the human microbiome, tend to harbor more complex and diverse RMS [53]. n such environments, the ability to recognize and cleave a broad range of foreign DNA sequences offers microbial populations a greater chance of protecting their genomes from the influx of harmful genetic material. Conversely, microbes living in more isolated or stable environments, such as endosymbiotic bacteria within host cells, tend to possess fewer RMS [54]. These bacteria, which experience limited exposure to external genetic threats, have a reduced need for complex defense systems, reflecting their relatively low risk of encountering foreign DNA. Furthermore, comparative genomics has unveiled not only the structural and functional diversity of RMS but also their varied genomic organization and regulation across different microbial taxa. Studies have demonstrated that RMS can range from simple, solitary loci to complex arrays of multiple RM systems, often organized into operons or multi-gene clusters [55]. These systems can be found in a variety of configurations, with some microbes encoding a single restriction enzyme paired with a corresponding methyltransferase, while others harbor multiple, distinct RMS loci, each with its own set of specificity determinants and regulatory mechanisms [37,56,57,58,59,60]. This modular organization allows for the fine-tuned regulation of RMS expression in response to environmental signals, such as the presence of foreign DNA or the activation of stress response pathways. The diversity of RMS is not solely a function of the number of systems present but also reflects their evolutionary adaptation to specific ecological niches and lifestyles. For instance, microorganisms that experience frequent interactions with foreign DNA, whether from bacteriophages or HGT, may evolve more specialized RMS to enhance their ability to discriminate between self and non-self DNA. On the other hand, microbes in more sheltered environments may retain simpler systems that are sufficient to protect their genomes against the occasional influx of foreign genetic material [56,61,62]. Moreover, the dynamism of RMS within microbial genomes is evident in the frequent genomic rearrangements, gene losses, and acquisitions observed in various microbial populations. The flexibility of RMS loci enables rapid adaptation to changing environmental conditions, including shifts in the prevalence of phages or the introduction of new genetic material via HGT [63]. These genomic fluctuations suggest that RMS are subject to intense selective pressures and are constantly being refined, discarded, or replaced in response to new challenges.

Therefore, comparative genomics has provided a comprehensive view of the diversity and evolutionary significance of RMS in microbial populations. These studies have revealed a complex landscape of RMS that varies not only in the number and specificity of systems present but also in their genomic organization, regulation, and adaptability to ecological niches. As microbial genomes continue to evolve in response to their environmental challenges, the diversity of RMS will likely expand, driven by mechanisms such as gene duplication, HGT and recombination, providing further insights into the intricate relationship between microbes and their genetic adversaries.

#### 2.2.3. Spectrum of RMS Across Microbial Species

Recent comparative studies have demonstrated that soil-dwelling bacteria, such as members of the genus *Bacillus*, possess a significantly higher number and diversity of RMS compared to bacteria isolated from relatively stable environments, such as endosymbionts [64]. In the human gut microbiome, analysis of *Bifidobacterium breve* has revealed unique methylation signatures associated with its RMS that potentially contribute to host-microbe interactions and competition [65]. Moreover, marine bacteria exhibit distinct RMS profiles that may be linked to the fluctuating nutrient conditions and high viral diversity in ocean ecosystems [66]. The spectrum of RMS across microbial species reveals the intricate ecological and evolutionary forces that shape these systems. Bacteria, for example, predominantly harbor Type II RMS, which are characterized by their high specificity and efficiency in recognizing and cleaving foreign DNA. These systems allow bacteria to effectively defend themselves against exogenous genetic elements, including bacteriophages and plasmids, by recognizing and cleaving invading DNA. In contrast, archaea often exhibit a preference for Type I and Type III RMS, systems that tend to be more complex in terms of regulation and mechanism of action. These systems are less specific than Type II, typically recognizing longer DNA sequences or requiring more elaborate multi-subunit complexes for their activity. The prevalence of Type I and Type III systems in archaea is thought to reflect their adaptation to extreme environments, such as high-temperature or highly saline habitats, where more sophisticated regulatory mechanisms for RMS activity may offer selective advantages [52]. In these environments, the ability to finely tune the expression and activity of RMS in response to environmental stresses, such as viral predation or horizontal gene transfer, can be a crucial factor in survival. The diverse strategies employed by bacteria and archaea highlight the evolutionary versatility of RMS, which have evolved to meet the unique challenges of different ecological niches.

The diversity of RMS extends beyond their enzymatic mechanisms to their regulation and integration within microbial genomes. Many microbes employ RMS as part of a larger, integrated network of genomic defense mechanisms [18,40,57]. A particularly striking example is the coexistence of RMS with CRISPR-Cas systems, where the two defense systems work in tandem to offer a multilayered defense strategy against genetic intruders. The CRISPR-Cas system provides adaptive, sequence-specific immunity through acquisition and utilization of short spacer sequences derived from previously encountered genetic threats. In contrast, RMS confer immediate and innate protection by recognizing and cleaving specific unmethylated foreign DNA sequences, thereby providing rapid, constitutive defense without prior exposure. Experimental evidence from *E. coli* and *S. thermophilus* demonstrates that these two defense mechanisms functionally complement each other, with RMS effectively restricting initial infection by unfamiliar bacteriophages, while CRISPR-Cas provides lasting protection against recurrent exposures through immunological memory [20,67,68]. For example, laboratory experiments in *S. thermophilus* showed that initial exposure to novel phages was primarily countered by RMS-mediated cleavage, but subsequent re-infections were rapidly neutralized by CRISPR-Cas using previously acquired spacers [69]. Such dual-layered defense significantly enhances microbial resistance to diverse and evolving genetic threats, highlighting a complex yet highly coordinated bacterial immune response. Recent work on *Pseudomonas aeruginosa* revealed that the expression of CRISPR-Cas systems can be actively modulated by the presence of RMS, indicating a sophisticated regulatory interplay. For example, the Type I-F CRISPR system in *P. aeruginosa* was found to exhibit decreased expression when certain RMS were highly active, suggesting that bacteria balance the metabolic and autoimmune costs associated with multiple simultaneous immune responses [70,71]. Moreover, the interplay between different genomic defense systems, such as RMS and CRISPR-Cas, reflects the ongoing evolutionary pressures that drive the diversification of these systems [72]. As microbial environments are subject to constant change, including the continual introduction of new genetic material through horizontal gene transfer and viral predation, the need for highly adaptable and efficient defense mechanisms has driven the co-evolution of multiple, complementary systems. In *Staphylococcus aureus*, detailed genomic analyses have shown that strains containing both the Sau1 Type I RMS and the Type III-A CRISPR-Cas system exhibit reduced horizontal gene transfer rates and increased resistance to phage predation compared to strains lacking one of these systems. Experimental infections with phages reveal that this dual-layered immunity significantly enhances bacterial survival by providing both immediate (RMS) and adaptive (CRISPR) protection, illustrating how these complementary mechanisms create a robust, multi-tiered defense [73,74]. This dynamic interaction between bacterial immune systems and bacteriophage infection pressures demonstrates how microbial populations rapidly evolve by altering defense strategies, such as modifying RMS specificity or adjusting CRISPR spacer acquisition rates. For instance, laboratory evolution experiments with *Pseudomonas aeruginosa* have shown rapid shifts in CRISPR-Cas spacer content in response to sequential phage challenges, exemplifying microbial adaptation to fluctuating selective pressures [75]. Beyond their individual functions, RMS also have ecological implications that extend to microbial community structure and dynamics. In environments characterized by high microbial diversity and intense competition, such as the human microbiome or soil ecosystems, RMS can mediate microbial interactions by conferring competitive advantages to their hosts. Furthermore, the regulation of HGT by RMS plays a critical role in the dissemination of genes that confer advantageous traits, such as antibiotic resistance, virulence factors, and metabolic capabilities [56,76,77]. While the coexistence of RMS and CRISPR systems provides bacteria with versatile immune capabilities, several fundamental questions remain unanswered. It is currently unclear how microbial cells prioritize immune responses when exposed to simultaneous pressures from multiple mobile genetic elements. Are regulatory networks generally conserved across different bacterial taxa, or are there lineage-specific variations reflecting unique ecological niches? Recent comparative genomic studies suggest significant diversity in regulatory architectures, underscoring the need for further experimental validation to elucidate the mechanistic basis for these differences [29,46,61,78]. Consequently, the diversity of RMS across microbial species is a reflection of the complex evolutionary forces that shape microbial defense systems.

### 2.3. Coexistence and Interplay of Multiple RMS Within Bacterial Species

A growing body of evidence indicates that many bacterial species do not rely on a single RM system but rather harbor multiple, coexisting systems that together create a multilayered defensive barrier. For instance, in *Staphylococcus aureus*, extensive genomic studies have revealed the simultaneous presence of several distinct RMS types. The Type I system Sau1, which exhibits lineage-specific variations, plays a critical role in limiting the uptake of foreign DNA and thus contributes to the clonal diversification seen among strains [79]. In parallel, *S. aureus* commonly possesses the Type IV system SauUSI, which specifically targets cytosine-methylated DNA, a feature frequently used by bacteriophages and mobile genetic elements to disguise themselves as “self” [80]. This dual action intensifies the restriction barrier, making HGT increasingly challenging and thereby affecting the evolutionary trajectory of the species. Beyond these, some strains, such as *Helicobacter pylori*, *Neisseria gonorrhoeae*, *Haemophilus influenzae*, and *Streptococcus pneumoniae*, even encode additional Type II and Type III-like RMS, which further complicate the genetic landscape by recognizing and cleaving distinct DNA sequences with their own unique recognition specificities and enzymatic properties [81]. The coexistence of these multiple systems can result in a synergistic or, in some circumstances, antagonistic interplay. For example, while one system may act as a broad-range barrier against diverse phage DNA, another may provide fine-tuned discrimination against specific genetic elements, collectively enforcing stringent control over the influx of exogenous DNA [82]. This multilayered defense ensures genome integrity by preventing the integration of deleterious mobile elements and restricting unwanted horizontal gene transfer events, yet it also poses significant challenges for genetic manipulation and recombinant DNA technologies. In *S. aureus*, multiple RMS coexist, creating a multilayered barrier to foreign DNA entry. Recent studies suggest intricate regulatory and functional dynamics among these systems, with evidence of coordinated expression to optimize immune efficiency and resource utilization. For example, the presence of active Sau1 (Type I) can influence the transcriptional regulation of Type IV systems like SauUSI, thereby modulating the restriction barrier according to environmental pressures [83]. The cumulative restriction activities provided by these multiple systems significantly reduce horizontal gene transfer and increase resistance to phage infection, emphasizing their combined protective effect. These complex interactions highlight an evolutionary strategy that balances immediate genomic protection with long-term adaptability, offering a compelling model for understanding microbial immune dynamics and genome evolution [84]. Thus, the coexistence and interplay of multiple RMS within a single bacterial species, exemplified by *S. aureus*, underscore the remarkable adaptability of bacterial defense strategies. This complex organization not only acts as a formidable barrier against HGT, thereby preserving genome stability and cellular integrity, but also drives evolutionary innovation by mediating selective pressures. Future research integrating advanced genomic and proteomic methodologies is expected to further elucidate the regulatory networks and mechanistic details underlying these complex interactions, providing deeper insights into bacterial physiology, evolution, and the challenges of genetic manipulation.

## 3. Biological Functions and Evolutionary Dynamics of RMS

RMS are a fundamental aspect of microbial life, deeply embedded in the evolutionary fabric of bacteria and archaea. Traditionally recognized for their role in defending against phage invasions, RMS have been increasingly acknowledged for their broader biological implications [85]. These systems not only serve as a primary line of defense against foreign genetic elements but also play important roles in maintaining genomic stability, regulating gene expression through epigenetic mechanisms, influencing microbial competition and community dynamics, and driving microbial evolution.

### 3.1. Phage Invasion and RMS Response

**Figure 4:**
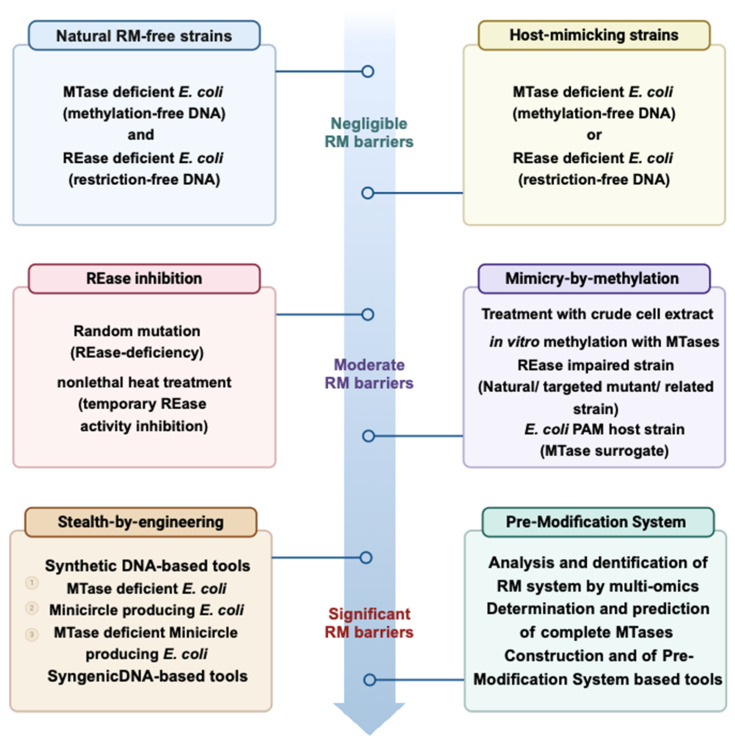
Strategies for overcoming genetic barriers in bacteria imposed by RM systems. Current methods either produce a methylation that is surrounded by the hosts endogenous (in vitro) or implanted methylome (ex vivo), with this functionally achieving some level of mimicry via methylation. SyngenicDNA approaches, on the other hand, circumvent RM systems by deleting their target recognition sequences from DNA to generate minimalistic (RM-silent) genetic tools and thereby implement stealth-by-engineering during transformation. The pre-modification system strategy involves using multi-omics analysis to fully investigate the RM expression profile of the desired strains. By introducing the cloning host strain and pre-modifying all RM recognition sites during plasmid construction, it rapidly achieves complete evasion of the RM barriers of desired strains.

Bacteriophages, with their vast and diverse genetic arsenal, present a persistent and ever-evolving threat to microbial populations, exerting significant selective pressure on host genomes. This defense mechanism, however, is not invulnerable. Over the course of coevolution, phages have developed a variety of countermeasures to bypass or evade the restrictive actions of RMS (Figure 4). One of the most common strategies employed by phages is the modification of their own genomic DNA to mimic the host’s methylation patterns [86,87,88]. For example, the *E. coli* phage T4 modifies its own DNA by methylating specific sites, thereby evading cleavage by the host’s restriction enzymes, such as EcoR1, that would otherwise target unmethylated DNA [89]. Similarly, the *Streptococcus* phage ϕ11 also uses DNA methylation to protect its genome from host restriction enzymes, specifically through the methylation of the recognition sites for the host’s restriction endonucleases [90]. This mimicry allows phages to evade the immune-like defense system of the host, ensuring successful replication and propagation within the bacterial cell. In some cases, phages also produce proteins that inhibit the activity of restriction enzymes, further enhancing their ability to evade host defense mechanisms.

The ongoing evolutionary “arms race” between phages and their bacterial hosts has driven the diversification of RMS across microbial genomes. The continuous development of novel phage evasion strategies has spurred the evolution of an ever-expanding repertoire of restriction enzymes with different specificities, enabling microbes to combat an increasingly diverse range of phage genotypes [91]. Consequently, bacterial genomes harbor a complex array of RMS, each tailored to recognize and cleave distinct sequences of foreign DNA, further enhancing microbial resilience against phage invasion [92]. The diversification of RMS is not limited to the development of new restriction enzymes but also includes the evolution of more sophisticated regulatory mechanisms that fine-tune the activity of these defense systems. Many bacteria possess additional layers of regulation that control the expression of RMS in response to environmental cues, such as the presence of foreign DNA or stress from phage predation. These regulatory circuits allow microorganisms to activate their defense systems only when necessary, thereby conserving energy and resources while providing protection against potential threats. Furthermore, some bacteria have evolved RMS configurations that enable the integration of multiple systems into a single, modular defense network [57,92,93]. This modularity allows for a more flexible and efficient response to phage invasion, enabling bacteria to respond rapidly to new or evolving phage populations.

Beyond the direct defense against phage infection, RMS also play an important role in modulating the genetic diversity of microbial populations. By cleaving foreign DNA during phage infections, restriction enzymes limit the horizontal transfer of genetic material, including virulence factors and antibiotic resistance genes. This can prevent the rapid spread of deleterious genetic traits within microbial communities, thereby contributing to the stability of microbial populations [94]. However, this regulatory function can also shape the genetic composition of microbial communities, influencing the evolution of microbial genomes and the dynamics of microbial competition. The interplay between phage evasion strategies and RMS diversification highlights the adaptability of both phages and their bacterial hosts. As phages continue to evolve novel methods to circumvent bacterial defenses, the microbial genome is likewise subject to constant evolutionary pressures that drive the refinement and diversification of RMS [18,19,95]. This coevolutionary process ensures that microbial populations are able to maintain robust defense systems in the face of evolving threats, while also contributing to the generation of genetic diversity that fuels evolutionary innovation. While these systems serve as a vital line of defense against phage invasion, the continuous evolution of phage countermeasures has led to the diversification of RMS, resulting in an expansive and varied repertoire of restriction enzymes with different specificities. This dynamic relationship between phages and RMS underscores the complexity of microbial immunity and highlights the broader implications of phage-microbe interactions in microbial evolution, ecology, and biotechnology.

### 3.2. Maintenance of Genomic Stability

RMS play a crucial role in maintaining genomic stability by preventing the integration of foreign DNA sequences into the host genome [96]. In environments characterized by high rates of horizontal gene transfer, such as those involving plasmid exchanges or bacteriophage predation, the role of RMS in safeguarding the host genome becomes particularly important. The methylation patterns introduced by MEases prevent the host’s own restriction enzymes from targeting its genomic sequences, thereby maintaining the integrity of essential genes and regulatory regions [81]. The specificity of this defense mechanism is vital to the maintenance of functional genome architecture, as it ensures that only foreign DNA, such as that of phages or plasmids, is targeted for degradation, while the host genome remains unharmed.

One notable phenomenon not previously discussed in depth is the post-segregational killing (PSK) mediated by certain RMS. In this context, many bacteria harbor plasmid-encoded RMS that maintain plasmid stability by killing cells which lose the plasmid upon cell division. When a cell fails to inherit the plasmid carrying the corresponding methyltransferase, the unprotected chromosomal DNA becomes susceptible to cleavage by the associated restriction endonuclease, leading to cell death [97]. This mechanism, also observed in the context of chromosomal RMS, reinforces the idea of “addiction systems” that ensure the maintenance of genetic elements within the cell. PSK has significant implications for understanding bacterial physiology, as it contributes to plasmid stability and imposes a selective pressure that influences both genome plasticity and evolutionary dynamics. Moreover, the interplay between PSK and HGT can shape the distribution of RMS among bacterial populations, ultimately affecting microbial adaptation and ecological fitness, which provides a more comprehensive view of the multifaceted roles of RMS in bacterial cell regulation and evolution [98].

RMS also contribute to genomic stability by combating the integration of mobile genetic elements, such as transposons and integrative plasmids. These elements, which can insert themselves into the host genome, pose a significant threat to genomic integrity, potentially disrupting essential genes or regulatory sequences. By selectively recognizing and cleaving these foreign DNA elements, RMS mitigate the risks associated with genomic instability and the introduction of harmful mutations [27]. This action is particularly important in preventing insertional mutations, which can result in gene disruption or altered regulatory networks that could impair cellular functions or drive evolutionary changes. Furthermore, the regulation of RMS activity is another critical aspect of their role in maintaining genomic stability. The expression and activity of both restriction enzymes and modification enzymes are finely tuned in response to environmental signals, such as the presence of foreign DNA [6]. Studies have shown that the modulation of RMS activity provides a dynamic mechanism for microorganisms to respond to varying levels of threat, such as fluctuating phage populations or the entry of new mobile genetic elements [15,99,100]. This adaptive regulation allows microorganisms to balance the need for defense against external genetic threats with the need to preserve their own genomic integrity.

The sophisticated nature of RMS is not merely that of static defense mechanisms but rather that of integral components of the microbial genomic regulatory network. By dynamically regulating their expression in response to environmental cues, RMS help to maintain genomic stability while allowing for evolutionary flexibility. This regulation provides microbes with the ability to fine-tune their immune-like responses and adapt to changing environmental conditions, ensuring the preservation of key genomic features while maintaining defense mechanisms against an ever-evolving array of genetic threats [57,63,101,102]. The importance of RMS in maintaining genomic stability extends to their role in regulating genomic plasticity. By controlling the influx of foreign genetic material, RMS influence the genetic composition of microbial populations, contributing to the preservation of beneficial traits while limiting the spread of potentially harmful elements. In this way, RMS act not only as defense mechanisms but also as modulators of microbial evolution, helping to shape the genetic landscape of microbial communities over time. Hence, RMS are integral to maintaining genomic stability in microorganisms. As fundamental components of the microbial genomic regulatory network, RMS play a critical role in safeguarding microbial genomes while enabling evolutionary adaptability in the face of continuous environmental challenges.

### 3.3. Genetic Regulation and Expression

The expression of restriction–modification (RM) genes is intricately regulated within microbial genomes, maintaining a delicate balance between the defensive effectiveness of the system and its associated metabolic costs. This fine-tuned regulation ensures that the expression of REases and MEases is modulated according to environmental signals, primarily the presence of foreign DNA. Such dynamic regulation prevents the unnecessary production of these enzymes, which could otherwise impose a significant metabolic burden on the host. This system’s ability to conserve energy and resources while providing an efficient defense mechanism is a testament to its evolutionary optimization [7]. Regulatory mechanisms governing RM gene expression operate at multiple levels, including transcriptional, post-transcriptional, and post-translational modifications [81].

Transcriptional regulation is typically mediated by repressor proteins, which can bind to the promoter regions of RM operons, preventing their transcription under non-inductive conditions. Conversely, the presence of foreign DNA, such as that from bacteriophages or plasmids, can trigger derepression, allowing for the activation of RM system expression. These responses are often regulated by complex networks of regulatory proteins and small RNAs that sense changes in the microbial environment and adjust gene expression accordingly [103,104]. Post-transcriptional regulation plays a key role in modulating RM system activity. For example, the stability and translation efficiency of RM mRNA can be influenced by RNA-binding proteins or regulatory RNAs, which ensure that the enzymes are synthesized only when necessary. This level of control allows for rapid adjustments in enzyme production in response to changing environmental conditions, such as DNA damage or the introduction of foreign genetic elements. In addition to transcriptional and post-transcriptional regulation, post-translational modifications further fine-tune the activity of RM enzymes [32,81,105,106]. These modifications, which include phosphorylation, acetylation, or proteolytic cleavage, can affect enzyme stability, activity, or interactions with other cellular components. Post-translational regulation ensures that RM enzymes are not only expressed in the correct quantity but also activated or inactivated in response to the specific needs of the cell. For instance, the modification of restriction enzymes by methylation or phosphorylation may alter their DNA cleavage activity or prevent the restriction of host DNA, thereby ensuring the protection of the host genome [6,103,107].

The regulatory complexity of RM systems reflects the adaptability of these systems to a wide range of physiological conditions and environmental threats. This adaptability is essential for the survival of microorganisms, as it enables them to respond rapidly to invading foreign DNA while minimizing the metabolic cost of producing enzymes that are not immediately needed. Additionally, RM system regulation plays a critical role in protecting the host genome from the potential damage that could result from inappropriate or excessive cleavage by restriction enzymes. Emerging studies have highlighted the role of RM gene regulation in microbial defense strategies beyond the classic concept of phage resistance [40,108,109]. Recent findings suggest that RM systems are involved in regulating HGT, which is a key mechanism for the spread of genetic material, including antibiotic resistance genes, among microbial populations [48,110,111]. By controlling the expression of RM enzymes, bacteria can selectively protect their genomes from the uptake of foreign DNA while allowing for the potential incorporation of beneficial genetic material. This regulatory role extends to the modulation of genomic plasticity, with RM systems contributing to the diversification and evolution of microbial genomes by restricting or promoting the integration of novel genetic elements.

In summary, the expression and regulation of RM genes are central to the efficient functioning of these systems, ensuring both effective defense against foreign DNA and metabolic efficiency. The sophisticated network of regulatory mechanisms that control RM gene expression at multiple levels underscores the system’s versatility and adaptability, enabling microorganisms to respond to various environmental challenges while minimizing the associated costs. Understanding the regulation of RM systems not only provides insights into microbial defense strategies but also opens up new avenues for biotechnological applications, including genome editing, synthetic biology, and antimicrobial development.

### 3.4. Evolutionary Origins and Adaptive Significance

The evolutionary origin of RMS is intricately tied to the dynamic and ongoing evolutionary arms race between microorganisms and bacteriophages. The selective pressure exerted by phages has been a driving force in the expansion and diversification of RMS, leading to the evolution of restriction enzymes with varied specificities and functions. This evolutionary battle has spurred the development of a diverse array of RMS components, each tailored to counter the ever-evolving strategies of viral predators. As a consequence, these systems represent a primordial form of microbial immune-like defense, finely honed over billions of years of coevolution between host and parasite [48]. Molecular phylogenetic studies have revealed that the components of RMS, particularly Type II restriction enzymes, have undergone significant horizontal gene transfer (HGT), facilitating their widespread distribution across both bacteria and archaea. Such HGT events have been pivotal in the global dissemination of RMS and have contributed to the extraordinary diversity of these systems [112]. This horizontal transfer, combined with gene duplication and functional diversification, has significantly shaped the evolution of RMS, reflecting the coevolutionary dynamics between microbes and their viral adversaries [113]. Phylogenetic analyses further support the hypothesis that RMS evolved from simpler, less specific ancestral forms into the highly specialized and diverse enzymes observed in contemporary microbial genomes [114,115,116]. The functional diversification of restriction enzymes through these processes highlights the remarkable adaptability of RMS in response to the ever-changing landscape of phage-driven selection.

The variability observed in RMS configurations across microbial genomes is another testament to the evolutionary strategies employed by microbes to enhance their genomic defense [117]. These systems range from relatively simple, solitary RM systems to highly complex arrays consisting of multiple systems, often integrated into larger genomic structures such as operons or multi-gene clusters. This modular nature of RMS evolution allows for flexibility in defense mechanisms, enabling microorganisms to rapidly adapt to new phage threats. The ability of these systems to evolve through recombination and the acquisition of novel specificity determinants underscores the remarkable adaptability of RMS in the face of viral evolution [7]. Moreover, the functional modularity of restriction enzymes, often facilitated by the exchange of enzyme subunits, enables rapid shifts in specificity, enhancing the ability of microorganisms to recognize and degrade an expanding variety of phage genomes. The evolutionary history of RMS also reflects their dual role in microbial survival [18,108,110]. First and foremost, RMS serve as a direct defense mechanism against foreign DNA, particularly the genetic material introduced by bacteriophages. By cleaving invading DNA, restriction enzymes protect the host genome from potential integration of phage DNA, which could otherwise disrupt cellular function or lead to viral replication. At the same time, the modification enzymes (MEases) methylate the host DNA, marking it as “self” and preventing it from being cleaved by its own restriction enzymes. This self/non-self discrimination mechanism is central to the function of RMS, highlighting the duality of these systems as both immune protectors and regulators of genomic stability.

In addition to their defensive role, RMS are crucial in shaping microbial evolution through their influence on HGT. By recognizing and cleaving foreign DNA, RMS regulate the influx of external genetic material, thereby influencing the genetic composition and evolution of microbial populations [91,93]. This selective control over gene acquisition plays a significant role in microbial adaptation, as it limits the integration of potentially harmful DNA while allowing for the incorporation of beneficial genetic traits. Consequently, RMS contribute not only to the defense against exogenous genetic threats but also to the modulation of genetic diversity within microbial populations [118]. This balance between defense and diversity is essential for microbial evolution, as it allows microorganisms to respond dynamically to environmental pressures while maintaining the integrity of their genomes. The ongoing coevolution between bacteriophages and their bacterial hosts has driven the refinement and diversification of RMS, resulting in a complex and adaptive defense system that plays an important role in microbial evolution [15]. Over time, the increasing specificity and complexity of RMS have allowed bacteria and archaea to fend off a broader spectrum of phage types and other foreign genetic elements [18,92]. This constant evolution of RMS, fueled by horizontal gene transfer, gene duplication, and functional divergence, underscores the remarkable ability of microbes to adapt to the relentless pressure exerted by phages and other genetic invaders.

In brief, the evolutionary origins of RMS are deeply rooted in the coevolutionary dynamics between bacteriophages and their microbial hosts. Through horizontal gene transfer, gene duplication, and functional diversification, RMS have evolved from simple, ancestral forms into highly specialized systems that provide both direct defense against foreign DNA and contribute to the genetic plasticity of microbial genomes. These systems not only serve as critical immune-like defenses but also play a crucial role in shaping the genetic composition and evolutionary trajectory of microbial populations, ensuring their survival in the face of continuous environmental challenges.

### 3.5. Evolutionary Dynamics of RMS Enzyme Families

The evolutionary trajectory of RMS enzyme families is a complex process driven by various selective pressures, including the arms race with bacteriophages and the dynamic interplay with other defense mechanisms such as CRISPR-Cas systems. To elucidate these processes, phylogenetic methods—such as maximum likelihood and Bayesian inference analyses—have been applied to trace the origins and diversification of RMS genes across diverse bacterial genomes [119]. These analyses reveal that HGT, gene duplication, and domain shuffling are key mechanisms underlying RMS diversification. For instance, comparative analyses of Type II restriction enzymes across different bacterial lineages suggest rapid evolution in their DNA-binding domains, likely driven by strong selection imposed by evolving phage populations [15]. Quantitative models of selection pressures, employing methods such as dN/dS ratio calculations, have further illustrated that certain RMS components are subject to strong positive selection, reinforcing the “arms race” dynamics [120].

Moreover, our analysis underscores the interplay between RMS and other bacterial defense systems. Notably, many bacteria co-harbor CRISPR-Cas systems alongside multiple RMS types. The coexistence of these systems suggests complementary roles: while CRISPR-Cas provides an adaptive immune response through the memory of previous phage exposures, RMS act as a constitutive barrier that offers immediate protection against invading DNA. Recent integrated studies have begun to quantitatively evaluate how these systems interact, showing that bacteria may regulate the expression of both defenses in a coordinated fashion to optimize resource allocation and minimize autoimmunity [121]. In addition, models that incorporate both ecological data and molecular evolution have illustrated that the combined selective pressures exerted by phage predation, HGT, and inter-system competition drive the continual refinement of bacterial defense strategies. These findings highlight that the evolution of RMS is not an isolated process; rather, it is intricately linked to the broader context of microbial defense, genome plasticity, and ecological adaptation. Future studies incorporating large-scale genomic, transcriptomic, and proteomic data sets are expected to further refine our understanding of these evolutionary dynamics and shed light on the relative contributions of different selection forces in shaping RMS diversity.

### 3.6. Anti-Defense Systems

Bacterial defense systems, such as RMS and CRISPR-Cas, exert significant selective pressure on bacteriophages and other mobile genetic elements. In response to these bacterial defenses, phages have evolved a range of anti-defense strategies that allow them to overcome or circumvent the host’s immune responses. This ongoing evolutionary “arms race” has far-reaching implications for our understanding of bacterial immunity and offers promising avenues for developing new strategies to manipulate bacterial populations. A common anti-defense mechanism employed by many phages is the modification of their own genomes. Phages may incorporate unusual bases or modify standard bases (e.g., through glycosylation or methylation) to mimic the host’s methylation patterns, thereby evading recognition and cleavage by host RMS. For example, the T4 phage is known to substitute hydroxymethylcytosine for cytosine, followed by further glucosylation, which effectively prevents cleavage by many host restriction enzymes [18]. Some phages encode proteins that directly inhibit bacterial restriction enzymes. These anti-restriction proteins—such as ArdA, ArdB, and Ocr—bind to restriction enzymes or their subunits to block the access of DNA substrates. This protein-based inhibition ensures that even if the host RMS is expressed, the phage genome is not recognized or degraded [122]. In parallel, bacteriophages have evolved anti-CRISPR proteins that neutralize CRISPR–Cas systems by binding to Cas proteins and inhibiting their DNA-cleavage activity. The discovery of diverse anti-CRISPR proteins has underscored a similar evolutionary pressure on adaptive immunity systems and has revealed new layers of complexity in phage–host interactions [123]. Another innovative strategy is DNA mimicry, where phages produce proteins that closely resemble the structure of DNA. These mimic proteins can competitively inhibit DNA-binding sites of restriction enzymes, essentially “decoying” the host’s defense machinery away from the authentic phage genome [124]. As research continues to uncover the molecular details of anti-defense systems, a more integrated view of bacterial immunity is emerging. Future studies combining genomics, proteomics, and structural biology are likely to provide further insights into how anti-defense systems modulate bacterial evolution and ecology. Additionally, the deliberate manipulation of these systems holds potential for novel therapeutic strategies, such as enhancing the efficacy of phage therapy against multidrug-resistant pathogens or engineering bacterial strains with customized defense capabilities.

## 4. Biotechnological Applications of RMS

Research on RMS has not just revealed key features of microbial defense mechanisms, but has also opened up possibilities for groundbreaking advancements in biotechnology and medicine. From the beginning of genetic engineering to the creation of new treatments, the uses of RMS highlight how microbial systems can add to scientific and technological progress.

### 4.1. Advances in RMS Characterization Techniques

Recent technological advances have profoundly improved our ability to study RMS and their associated methylation patterns. Among these, Single-Molecule Real-Time (SMRT) sequencing has emerged as a powerful tool for methylome analysis. SMRT sequencing not only provides long-read sequencing data but also directly detects DNA modifications by measuring variations in the kinetics of DNA polymerase during synthesis. These kinetic signals allow for the detection of methylated bases at single-molecule resolution, thus enabling researchers to map methylation patterns across complete genomes and identify active RMS [125]. In addition to SMRT sequencing, other methods, such as nanopore sequencing and bisulfite sequencing, have been employed to assess DNA modifications. Nanopore sequencing, in particular, offers the advantage of real-time analysis and can discriminate multiple types of base modifications directly from DNA strands [126]. Combined, these techniques have expanded our understanding of the distribution, dynamics, and biological roles of RMS in microbial genomes, providing insights into both their evolutionary history and regulatory networks. The incorporation of these modern methods has not only enhanced the accuracy of methylome analysis but also facilitated comparative genomic studies that reveal the ecological and evolutionary significance of RMS across diverse bacterial species.

### 4.2. Gene Editing and Molecular Cloning

The emergence of Type II restriction enzymes in the 1970s signaled the beginning of modern genetic engineering [96,127]. RMS have played an essential role in the development of classical molecular cloning techniques. For example, Type II enzymes such as EcoRI and BamHI have long been used to construct recombinant plasmids and genomic libraries, thereby facilitating gene cloning and sequencing projects [128]. The technique of molecular cloning involves using restriction enzymes to create DNA fragments and ligases to connect these fragments into plasmids or vectors for insertion into host organisms [104]. This method has played a key role in genome mapping, gene function research, and recombinant protein production. Cohen et al. (1973) showed how restriction enzymes were used to create recombinant plasmids, revolutionizing the field of genetic engineering [129]. Ever since then, there has been rapid advancement in the field, with RMS being key in the development of technologies like CRISPR-Cas9, which, although different from RMS, also focuses on modifying nucleic acids based on specific sequences. One of the deepest impacts of RMS has been seen in the areas of genetic editing and molecular cloning. The identification of Type II restriction enzymes, which cleave DNA at precise recognition sites, has played a crucial role in the advancement of recombinant DNA technology. This advancement allowed for the accurate removal and addition of DNA fragments, making it easier to clone genes, build genomic libraries, and develop genetically modified organisms (GMOs) [35,130]. A new method has been developed to avoid restriction–modification systems in bacteria by analyzing the genetic and methylation patterns of a specific bacterial strain, identifying the bacteria’s target motifs, and creating a customized “SyngenicDNA” tool for transformation, improving transformation efficiencies in species like *S. aureus* [131]. This strategic manipulation emphasizes the remarkable progress in microbial genetics, potentially paving the way for innovative bacterial genetic engineering without the limitations of restriction–modification barriers.

However, despite these successes, current applications face several limitations. Many RMS-based techniques are constrained by off-target effects, the inherent complexity of multi-subunit enzyme systems, and challenges in precisely modulating gene expression, which can restrict their broader use in industrial-scale processes. The specificity of RMS can sometimes be a double-edged sword, as natural variations in recognition sequences may lead to incomplete or aberrant cutting. Additionally, the rigidity of some RMS-based methods can limit flexibility when dealing with highly variable genomes or when introducing large genomic alterations. Furthermore, while RMS methods are invaluable in academic research settings, their translation to clinical or commercial applications is often limited by cost, regulatory hurdles, and the need for further optimization in terms of efficiency and specificity. These limitations underscore the necessity for continuing research to refine RMS-based technologies before they can be widely adopted in practical applications.

### 4.3. Synthetic Biology and Metabolic Engineering

Synthetic biology aims to construct new biological parts, devices, and systems or redesign existing ones to address specific needs in biotechnology. The ability to engineer microbial cells for the production of valuable compounds or the development of novel biosynthetic pathways depends critically on the precision with which genetic material can be inserted, deleted, or modified. Traditional methods of genetic engineering often rely on methods such as homologous recombination or CRISPR/Cas, but these approaches may not always be sufficient when higher specificity or more precise control is needed, particularly in complex pathways or high-throughput applications. RMS, and, in particular, Type II REases, provide a highly specific and effective means of achieving genetic modification. These enzymes, which cleave DNA at defined sequences, are essential tools in molecular cloning, enabling the precise cutting and pasting of genetic material [33,132]. The precision of RMS can be harnessed to build complex genetic circuits, integrate synthetic biosynthetic pathways into microbial hosts, or even develop systems for gene drive applications, allowing for the precise and predictable modification of microbial genomes. Furthermore, the modularity of RMS enables their adaptation to diverse synthetic biology applications. The widespread availability of commercially produced REases, which target a vast array of DNA sequences, allows for the easy customization of genetic engineering protocols to suit the specific needs of a given project. This flexibility is particularly beneficial in metabolic engineering, where the precise integration of biosynthetic genes into microbial hosts is required for the efficient production of target compounds [133]. As synthetic biology continues to evolve, the ability to leverage the diversity of RMS for genome manipulation will likely play a central role in the development of novel biotechnological applications.

Metabolic engineering, which focuses on the optimization and redesign of microbial metabolic pathways for the production of biofuels, pharmaceuticals, and specialty chemicals, relies heavily on the ability to manipulate microbial genomes in a controlled and reproducible manner [134]. In this context, RMS offer advantages in optimizing and expanding the range of available metabolic pathways within host organisms. The use of RMS in metabolic engineering has proven potential for improvements in microbial strain performance. By using REases to cut and integrate genetic material at precise locations within the host genome, scientists can build strains that produce high yields of biofuels such as ethanol, butanol, or biodiesel [135]. Similarly, RMS can be applied in the development of strains capable of producing pharmaceuticals, including antibiotics, enzymes, and even complex therapeutic proteins. One example of this is the use of RMS to create engineered strains of *Escherichia coli* that can efficiently produce high-value bio-based chemicals by precisely inserting the necessary biosynthetic genes into their genomes [136,137,138]. In these cases, RMS offer a level of control that is often more difficult to achieve with other methods, providing higher specificity, reduced off-target effects, and increased efficiency in pathway integration. Additionally, RMS contribute to the field of synthetic biosensors and regulatory circuits, which are fundamental components of engineered metabolic networks. For instance, the incorporation of REases into synthetic gene regulatory networks can be used to create highly sensitive biosensors that detect the presence of specific metabolites or environmental changes [139,140,141,142]. By integrating these biosensors into microbial systems, metabolic engineers can develop self-regulating systems that optimize the production of desired compounds, such as biofuels or pharmaceuticals, in response to fluctuating environmental conditions or nutrient availability.

### 4.4. Pharmaceutical Development, Diagnostics, and Gene Therapy

The potential applications of RMS in pharmaceutical development, diagnostics, and gene therapy are increasingly recognized as pivotal in advancing molecular medicine. RMS, which function to protect microbial genomes from foreign DNA through the combined action of REases and MEases, are not only essential for microbial defense but also offer a range of biotechnological opportunities. The precision with which restriction enzymes cleave DNA at specific sequences makes them invaluable for the development of recombinant DNA technologies, which are at the heart of many modern drug production processes [143,144,145]. By facilitating the construction of expression vectors and optimizing the integration of foreign genes into host genomes, RMS contribute to the generation of microorganisms that can produce high yields of therapeutic proteins, monoclonal antibodies, and vaccines. For example, *Escherichia coli*, *Saccharomyces cerevisiae*, and mammalian cell lines are commonly used as expression systems for recombinant proteins, with RMS playing an important role in the integration of therapeutic genes into these hosts. REases can be used to cut genomic DNA at precise locations to allow for the integration of foreign genes, facilitating the production of recombinant therapeutic proteins such as insulin, erythropoietin, and growth factors [17,146]. Additionally, RMS provide a mechanism to prevent the degradation of these recombinant products by ensuring that only foreign, non-methylated DNA is targeted for cleavage, while the host genome is protected by its native methylation patterns. Restriction enzymes have traditionally been utilized in constructing genomic and cDNA libraries, providing foundational tools for molecular cloning and functional screening. Although these techniques are now typically integrated with advanced sequencing technologies, such as NGS, they remain valuable in high-throughput screening contexts for drug discovery.

In molecular diagnostics, RMS have found significant applications in the detection and characterization of pathogens, genetic disorders, and cancer mutations. The high specificity of restriction enzymes for particular DNA sequences makes them ideal for diagnostic applications, where precise identification of genetic material is crucial. One of the most common uses of RMS in diagnostics is in the detection of pathogenic DNA, such as that from bacteria, viruses, or other microorganisms [5,17,147,148]. Restriction Fragment Length Polymorphism (RFLP), a classic molecular technique employing restriction enzymes combined with PCR and gel electrophoresis, is routinely utilized for precise identification and characterization of microbial pathogens and genetic variants associated with diseases. For example, RFLP is a well-established technique for bacterial strain typing, such as in the case of *Mycobacterium tuberculosis*, *Streptococcus pneumoniae*, and *Escherichia coli* [149]. These techniques are often used in conjunction with polymerase chain reaction (PCR) and gel electrophoresis to rapidly identify the presence of pathogenic DNA. RMS also play a role in the detection of genetic mutations that underlie various diseases, including inherited genetic disorders and cancers. By using restriction enzymes to cleave genomic DNA from patients at specific mutation sites, diagnostic tests can identify variations in the genome that are associated with diseases such as cystic fibrosis, sickle cell anemia, and certain types of cancer [5,150,151]. In cancer diagnostics, RMS are used to identify genetic mutations or epigenetic changes that drive tumorigenesis. By analyzing the methylation patterns of tumor DNA or detecting mutations using restriction enzymes, clinicians can develop better diagnostic tests to identify cancer subtypes, predict prognosis, and monitor treatment response. The ability of RMS to distinguish between self and non-self DNA has implications in the detection of tumor-specific DNA markers, which can aid in non-invasive cancer screening methods, such as liquid biopsies [5,152].

Gene therapy, which aims to treat or prevent disease by modifying the genetic material of a patient’s cells, represents one of the most promising therapeutic approaches in modern medicine [153]. Restriction–modification systems offer a precise mechanism for genome editing, allowing for the targeted modification of genetic sequences in both somatic and germline cells. The role of RMS in gene therapy is particularly significant in the context of genetic diseases caused by mutations in specific genes. By using REases to cleave DNA at precise loci within the genome, RMS can facilitate the insertion, deletion, or modification of genes with remarkable specificity. One of the key advantages of using RMS in gene therapy is their ability to provide targeted cleavage at specific DNA sequences, which is critical for the accurate integration of therapeutic genes or the correction of genetic mutations. In contrast to broad-spectrum genome editing techniques like CRISPR/Cas, which can sometimes result in off-target effects, RMS can be used to generate more predictable and precise edits in the genome, minimizing the risk of unwanted genetic changes [53,72,147]. This precision is particularly valuable in gene therapies aimed at treating genetic disorders, such as cystic fibrosis, muscular dystrophy, or hemophilia, where the insertion of a corrective gene into a specific location is required for therapeutic success. RMS also contribute to the development of gene delivery systems. The integration of foreign genes into the host genome, facilitated by the action of REases, is a critical step in many gene therapy protocols. By using RMS to integrate therapeutic DNA into the host genome in a controlled manner, gene therapy can be performed more efficiently, with a higher success rate [76,152]. Furthermore, the ability to use RMS in conjunction with viral vectors or other delivery systems allows for the efficient and stable expression of therapeutic genes, further enhancing the potential of gene therapy as a treatment modality.

### 4.5. RMS in Environmental Biotechnology

Microbial communities play a critical role in environmental processes, such as the degradation of organic matter, nutrient cycling, and pollutant degradation [154]. The functional diversity of these communities, driven by a wide variety of microbial species, is essential for their effectiveness in environmental bioremediation. RMS contribute to the regulation of microbial genome stability and diversity, offering a means to engineer microbial communities with specific functional traits for applications such as bioremediation, bioaugmentation, and biosensing. One of the most promising applications of RMS in environmental biotechnology is in the engineering of microbial communities for targeted pollutant degradation. By using RMS to selectively integrate genes encoding enzymes involved in the degradation of toxic compounds (e.g., heavy metals, pesticides, and industrial pollutants), scientists can enhance the capability of microbial strains to degrade or detoxify hazardous substances. Furthermore, RMS can help to regulate the expression of these genes, ensuring optimal functionality and stability of engineered strains over time [104,155,156]. This ability to modulate genetic expression, while protecting the host genome, is crucial for maintaining the long-term effectiveness of engineered microorganisms in environmental applications. The regulation of gene flow through horizontal gene transfer is another area where RMS can be harnessed in microbial community engineering. By controlling the movement of foreign DNA into microbial populations, RMS can prevent the spread of genetic elements that might compromise the stability or integrity of engineered communities [157].

Bioremediation, the use of microorganisms to degrade or neutralize environmental contaminants, is one of the most well-established applications of environmental biotechnology. RMS have a direct impact on bioremediation efforts by providing a means to enhance the genomic stability and adaptability of microbial strains used in these processes [154,157,158]. The introduction of foreign genes involved in pollutant degradation, detoxification, or resistance mechanisms into microbial strains is often required for the efficient breakdown of complex contaminants. Restriction enzymes, through their ability to recognize and cleave foreign DNA, allow for precise integration of these genes into microbial genomes, ensuring efficient expression and long-term stability. In the context of bioremediation, RMS can be used to engineer microorganisms capable of degrading complex pollutants such as petroleum hydrocarbons, chlorinated solvents, and pesticides. For instance, certain bacteria are capable of metabolizing hydrocarbons in oil spills or degrading pesticides in contaminated agricultural soils [159,160,161]. By using RMS to insert, delete, or modify genes related to the degradation of these compounds, microbial strains can be optimized for better performance. Additionally, RMS can help ensure that introduced genes remain integrated and functional within microbial populations, reducing the risk of gene loss or instability over time. This precision makes RMS an invaluable tool for improving the efficacy of bioremediation in diverse environmental conditions. The use of RMS in combination with other biotechnological tools, such as CRISPR-based genome editing or synthetic biology approaches, holds significant potential in enhancing the specificity and efficiency of bioremediation strategies. For example, engineered microbes equipped with biosensors—using RMS to control the expression of specific genes in response to contaminants—can be used to detect and degrade pollutants more effectively [148,162,163]. These engineered microbes could be deployed in areas such as contaminated water bodies, industrial effluents, or landfills, where conventional methods of pollution control are less effective.

RMS are also being explored for their potential in the treatment of waste materials, including the degradation of organic matter and the removal of heavy metals or other toxic substances from wastewater. The microbial degradation of organic waste is a critical process in composting and wastewater treatment systems, and RMS can play an important role in optimizing these processes. By regulating the microbial genomes involved in these processes, RMS can enhance the efficiency of microbial degradation pathways, leading to more effective treatment systems [99,139,164]. In wastewater treatment, RMS can be used to modify microorganisms for the bioremediation of pollutants, such as nitrogen, phosphorus, and heavy metals, which are commonly found in industrial wastewater. By integrating genes involved in the detoxification or removal of these contaminants into the microbial genome using RMS, microbial strains can be engineered for more efficient pollution control. Additionally, RMS can be used to develop biosensors for monitoring pollution levels in real-time, providing valuable information for the optimization of wastewater treatment processes. The role of RMS in pollution control is not limited to the degradation of organic compounds or the removal of heavy metals. RMS are also crucial in the prevention of the spread of antibiotic resistance genes in environmental systems. In environments where antibiotics are commonly used, such as agricultural settings or wastewater treatment facilities, the spread of antibiotic-resistant genes can pose a significant threat to both environmental and human health [11,12,14]. By using RMS to regulate the flow of resistant genes and prevent their dissemination, it is possible to mitigate the environmental impact of antibiotic resistance.

## 5. Perspectives and Future Directions

One major challenge is the genetic tractability of bacteria. As microbes evolve to evade RMS by altering recognition sites or acquiring novel methyltransferase activities, researchers must continually adapt their approaches. This evolutionary arms race underscores the need for dynamic RMS research that integrates cutting-edge bioinformatics and molecular biology to predict bacterial responses and engineer more robust systems [10]. Another significant concern involves ethical and safety considerations in deploying RMS for genetic engineering. Given the transformative potential of genome-altering technologies, it is imperative to establish stringent regulatory frameworks and ethical guidelines to ensure that RMS applications do not pose unforeseen risks to the environment or human health. A further challenge in harnessing RMS for broader applications lies in managing their specificity and mitigating off-target effects. While the precision of restriction enzymes enables targeted DNA manipulation, any deviation in recognition sites can result in unintended genomic alterations [165]. This highlights the necessity for a deep understanding of microbial genomic architecture and the development of advanced computational tools to predict and minimize off-target risks.

Despite these challenges, the future of RMS in biotechnology is highly promising, driven by rapid advances in genome editing, synthetic biology, and systems biology. RMS-mediated genome editing techniques offer unprecedented opportunities for microbial genome manipulation with high efficiency and stability [10]. The ability to achieve scarless genetic modifications across diverse microorganisms has the potential to revolutionize medicine, agriculture, and environmental science. For instance, a novel strategy that involves sequencing the genome and methylome of a bacterial strain to define its RM target motifs, followed by the synthesis of an RM-silent “SyngenicDNA” tool, demonstrates a systematic approach to evade RMS during transformation [131]. Such strategic manipulation may ultimately pave the way for bacterial genetic engineering that circumvents the constraints imposed by restriction–modification barriers (Figure 4).

The development of innovative biotechnological applications, such as the highly efficient production of marker-free transgenic lines, underscores the transformative impact of RMS on fields ranging from biopharmaceuticals and crop protection to animal health [166]. Moreover, the inherent mobility of RMS within genomes exemplifies their dynamic nature and significant potential for advancing genetic research and bioengineering [42]. Recent advances in genomics and synthetic biology offer unprecedented opportunities for RMS in industrial biotechnology, enabling the creation of novel biocatalysts and bio-tools for efficient and cost-effective bioconversion processes [167]. Additionally, the incorporation of noncanonical amino acids and the expansion of the genetic code are paving the way for the generation of synthetic organisms with altered chemical properties [168]. Although our understanding of RMS diversity has been greatly enhanced by genomics, bioinformatics, and molecular biology, many questions remain unanswered. Future research must focus on the functional characterization of novel RMS, the elucidation of regulatory networks governing their expression, and a comprehensive analysis of their biological roles and ecological impacts. Integrating genomic, transcriptomic, and proteomic data into large-scale models that accommodate diverse, functionally orthologous sequences will be crucial for advancing this field [147]. Moreover, exploring the interplay between RMS and other immune-like defense systems such as CRISPR-Cas is essential for achieving a holistic understanding of microbial defense, particularly in marine environments [169,170]. Ultimately, harnessing the full range of RMS as a biological resource holds promise not only for genome editing and synthetic biology but also for the development of novel antimicrobial drugs.

And, as research in RMS develops further and the significance of its promising applications is realized. Frontiers to Probe Exciting new areas of discovery comprise nanotechnology (where RMS could potentially build DNA-based nanostructures) and neurogenetics, where gene editing opens possibility for novel treatments in neurological disorders [152,171]. Nonetheless, these advancements pose concomitant sets of challenges as well; the most primarily being linked to ethical considerations and mechanisms assuring biosafety while employing RMS-based technologies. Economic growth will depend on these regulations walking the fine line between supporting innovation and safeguarding safety, privacy, ethics as RMS technologies are adopted into healthcare, agriculture and industry. In addition, the aim of public interaction and education would be to deal with societal issues while creating open-minded dialogues about the benefits of these technologies and their risks. Research into RMS continues to expand, and ongoing studies are expected to further elucidate their biological functions and enhance their practical applications. As scientists continue to develop ways for GMOs to optimize agricultural practices, there are ethical concerns related to gene editing and the potential risks of these organisms in complex biological systems. RMS are legacies, which represent some of the earliest and most basic RNA-mediated bacterial defenses against foreign DNA that continue to be adapted into genome editing tools or used for microbial genome engineering, as well as applications in biopharmaceutical production, crop protection, and more.

The integration of RMS into biotechnological applications extends to the high-efficient production of marker-free and uniform transgenic lines, crucial for applications in animal health, biopharmaceutics, and crop protection [172]. This underlines the potential of RMS in revolutionizing biotechnological applications, where precision and efficiency are paramount. In addition to genomic manipulation, RMS can potentially be applied in biotechnology for the isolation and amplification of new nucleic acid molecules, enabling the positive regulation and specific sequences identification for the production of amplification products [14]. Advances in genomics, systems biology, and synthetic biology further leverage the capabilities of RMS in industrial biotechnology, enabling the development of novel biocatalysts and bio-tools for efficient biological conversion processes [167]. The incorporation of noncanonical amino acids by expanding the genetic code showcases the synthetic biology’s potential in transferring chemical functionalities from the laboratory into the cellular environment [173].

Lastly, the application of the iEditing device in biotechnology demonstrates the rapid and efficient genomic engineering capabilities in electroactive bacteria, enhancing environmental applications through improved extracellular electron transfer systems [174]. The novel iEditing device for programming versatile extracellular electron transfer showcases the role of RMS in promoting sustainable and eco-friendly solutions [174]. This highlights the role of RMS in environmental biotechnology, contributing to sustainable and eco-friendly solutions. Further emphasizing the utility of RMS in biotechnology, a study showcases the RMS-mediated genome editing technique’s universal usefulness for microbial genome manipulation. This approach enables high-efficiency and stability in scarless genetic manipulations across various microorganisms [10], positioning RMS as a cornerstone for microbial engineering endeavors. This unique mechanism has laid the foundation for numerous biotechnological applications, from genetic engineering to the development of novel therapeutics.

## 6. Conclusions

From their origins as bacterial defense mechanisms to their integration into cutting-edge biotechnological applications, restriction–modification systems (RMS) have undergone a transformative journey. The extensive functional diversity of RMS offers both exciting opportunities and formidable challenges, shaping future directions in synthetic biology and genome editing, particularly in non-model bacterial strains. By elucidating the intricate molecular mechanisms underlying RMS function, researchers are poised to unlock novel medical therapies, promote sustainable agricultural practices, and enhance environmental protection strategies. Moreover, the pursuit of genetic tractability and the need for high-precision RMS deployment—encompassing accurate targeting and controlled expression—have spurred continuous innovations in RMS methodology. These advancements are critical to ensuring both the efficacy and safety of RMS applications across diverse biotechnological domains.

## Figures and Tables

**Table 1:** Representative features of typical RM systems in bacteria.

RM Type	Rease ^a^	MTase ^b^	Recognition Sequence	Modification Base	ATP Requirement	Notable Features	References
I	EcoKI	M.EcoKI	AACNNNNNNGTGC	m6A	ATP	Multi-subunit complex (HsdR, HsdM, HsdS); cleaves DNA at distant sites from the recognition sequence	REBASE ^c^
I	EcoAI	M.EcoAI	GAGNNNNNNTC	m6A	ATP	Multi-subunit complex; typical Type I system	REBASE ^c^
I	EcoBI	M.EcoBI	GGCCNNNNN	m5C	ATP	Multi-subunit complex; exhibits complex DNA recognition mechanisms	REBASE ^c^
II-A	EcoRI	M.EcoRI	GAATTC	m6A	None	Produces 5′ overhang; classical enzyme used in cloning	REBASE ^c^
II-A	HindIII	M.HindIII	AAGCTT	m6A	None	Produces sticky ends; widely used in genome engineering	REBASE ^c^
II-A	BamHI	M.BamHI	GGATCC	m6A	None	Frequently used in construction of expression vectors; produces sticky ends	REBASE ^c^
II-A	SmaI	M.SmaI	CCCGGG	m5C	None	Produces blunt ends; highly specific cleavage	REBASE ^c^
II-A	NcoI	M.NcoI	CCATGG	m5C	None	Produces blunt or sticky ends (depending on conditions); common in subcloning	REBASE ^c^
II-A	PstI	M.PstI	CTGCAG	m6A	None	Produces sticky ends; used in genomic library construction	REBASE ^c^
II-A	KpnI	M.KpnI	GGTACC	m6A	None	Produces sticky ends; widely used in subcloning	REBASE ^c^
II-B	NgoMIV	M.NgoMIV	GCCGGC	m5C	None	Belongs to Type II-B enzymes; exhibits unique recognition specificity	REBASE ^c^
II-S	BsaI	M.BsaI	GGTCTCN_1_	m5A	None	Type IIS enzyme generating defined overhangs; frequently used in DNA assembly	REBASE ^c^
II-S	BsmI	M.BsmI	GAATGCN	m6A	None	Precise cleavage activity; commonly used in molecular cloning	REBASE ^c^
II-S	AlwI	M.AlwI	GGATCN_4_	m6A	None	Shares recognition sequence with BamHI but has a different cleavage mechanism	REBASE ^c^
II-M	DpnI	–	GATC	–	None	Type IIM enzyme; cleaves only methylated DNA; no cognate methyltransferase	REBASE ^c^
II-I	DpnII	M.DpnII	GATC	m6A	None	Recognizes unmethylated DNA; usually functions in conjunction with its cognate methyltransferase (M.DpnII)	REBASE ^c^
II-M	–	M.SssI	CGCG	m4C	None	CpG Methyltransferase; no cognate restriction endonuclease	REBASE ^c^
II-G	BspMI	M.BspMI	ACCTGCN_4_	m6A	None	Produces sticky ends via a specific cleavage site; used in specialized cloning applications	REBASE ^c^
II-S	FokI	M.FokI	GGATG	m6A	None	Widely applied in gene editing; its cleavage domain is used in engineered nucleases	REBASE ^c^
III	EcoP15I	M.EcoP15I	CAGCAG	m6A	ATP	Type III enzyme; cleaves approximately 25 bp from the recognition site	REBASE ^c^
III	EcoP14I	M.EcoP14I	CAGCAG	m5C	ATP	Similar to EcoP15I but with different modification specificity	REBASE ^c^
IV	Mrr	–	non-specificity	non-specificity	None	Type IV enzyme; exhibits broad non-specific restriction activity	[23]
IV	McrBC	–	G^m5^CGC	m5C	GTP	Recognizes methylated DNA; effective against phage DNA	[10]
IV	McrA	–	Y^m5^CGR	m5C	None	Recognizes specific methylated modifications	[24]
IV	McrC	–	GGW^m5^CC	m5C	None	Works in cooperation with McrA; enhances recognition of methylated DNA	[25]
IV	MrrI	–	non-specificity	non-specificity	None	Similar to Mrr; targets non-specifically modified DNA	[26]
IV	MmeI	–	TCCRA^m5^C	m5C	None	Possesses both restriction and modification functions; often used in genome editing	REBASE ^c^
IV	MboII	–	G^m6^ATC	m6A	None	Acts on methylated DNA; part of phage defense mechanisms	REBASE ^c^
IV	MspI	–	C^m5^CGG	m5C	None	Sensitive to methylation status; commonly used in DNA methylation analysis	REBASE ^c^
IV	CviJI	–	CC^m6^ANNNNNNTGG	m6A	None	Recognizes modified sequences; frequently applied in studies of DNA methylation patterns	REBASE ^c^

^a^ REase, restriction enzyme; ^b^ MTase, modification enzyme; ^c^ REBASE, http://rebase.neb.com/rebase/rebase.html (accessed on 10 April 2025).

## Data Availability

No new data were created or analyzed in this study. Data sharing is not applicable to this article.
